# Standards of Excellence and Open Questions in Cancer Biomarker Research: An Informatics Perspective

**Published:** 2007-02-28

**Authors:** James Lyons-Weiler

**Affiliations:** University of Pittsburgh Cancer Institute, Pittsburgh, USA

Prospective authors and readers: welcome to *Cancer Informatics*!

In these pages, we hope to facilitate a global comprehension of advances in bioinformatics, computational biology, statistics, pathology informatics, and software design and engineering in support of cancer research and clinical practice.

As the founding Editor-in-Chief of *Cancer Informatics*, my first duty is to thank the Editorial Board for their support and participation, and their input on the direction, scope and focus of the journal, and also and especially Tim Hill. Without their considerable generosity and personal effort and passion in launching the journal, *Cancer Informatics* would not exist. I also gratefully acknowledge Dr. William Grizzle’s critical role as co-editor of this volume, and for overseeing the independent review process for two submitted manuscripts (Normelle et al & Lyons-Weiler et al).

As Editor-in-Chief, I am naturally tasked with the honor and responsibility of helping to navigate the direction and focus of the journal. In consultation with our world-class Editorial Board, we have, by design, been broad in our consideration of what the focus of articles would be acceptable for *Cancer Informatics*. In a nutshell, *Cancer Informatics* will publish papers that describe improvements in methods of computing that promote or facilitate excellence in cancer research and clinical practice.

Our readers can expect to find a distillation of papers that reflects, to a degree, the larger literature on bioinformatics, computational biology, and biomedical informatics that appear to have a high likelihood of promoting excellence in cancer research and clinical practice. They can also expect to find contributions that represent advances in biomedical informatics specialized for oncology, pathology and radiology. Individual papers and ideas on themed volumes (such as focused conference proceedings) in any of the areas described in the mission statement are quite welcome.

Our scope and focus are intentionally very broad, and not at all restricted to biomarker analysis. This is by design so as to avoid a myopic representation of Cancer Informatics. Our inaugural volume is dedicated to the problem of discovering, evaluating and validating cancer signatures in proteomic peptide profiles. The papers were drawn mostly from an EDRN (Early Detection Research Network) Proteomics Bioinformatics symposium held in Seattle, WA (March 2004). Each paper was subjected to careful peer review, and some papers presented at that meeting not appearing in this volume are still under revision as a result of the stringency of that review process.

In a recent survey (Auer et al., 2005), the number one need identified by researchers who use microarrays was ‘bioinformatics support’. This certainly includes data management, but it primarily references the need for advances in easy to use, easy to understand robust methods of analysis that lead to meaningful basic and applied discoveries.

Let’s consider each of these three criteria closely:

**Easy to use:** this means different things to different people. To the microarray facility manager and cancer researcher, it means ‘no programming’. Bioinformatics represents a bottleneck in throughput. The answer to opening up the bottleneck is not merely neat algorithms, but people; personnel trained to use bioinformatics analysis packages, and especially trained in the area of comparative evaluation of method of analysis. Such people may or may not program, but a workforce of data analysts is needed who know how to think critically about the relative performance of methods for finding biomarkers in a haystack, for rendering sample classifications, and for transforming results from a data set into an intelligible summary. ‘Easy to use’ to developers means ‘programmable API’. Biostatisticians, for example, are fond of using the R programming language, and many find ‘black box’ solutions anathema. The existing biostatistics workforce is insufficient both in numbers, and in their predilection to overtly compare the performance of various methods. Therefore, ‘easy-to-use’ has a dual meaning depending on the intended adopter.

**Easy to understand:** methods of analysis that cannot be easily understood by most cancer researchers can be compared to simpler methods via comparative evaluation, specifically the use of independent test validation sets. There are practical limitations that may impede the translation of disease prediction models; for example, output from models that cannot be understood by practioners on a pathology report may not be widely acceptable in clinical settings. In any case, it is the responsibility of the authors to explain their methods clearly, and it is the reviewer’s responsibility to inform the author on ways to improve clarity; each published paper is an opportunity for educating cancer researchers, and other informaticians. Contributing authors are therefore encouraged to write their papers accordingly by taking the time and space needed to explain concepts that might be foreign to people with different professional backgrounds.

**Appropriately designed:** cancer researchers should hope that the methods of analysis used are designed for large dimensional (many parameter, or large p) and small sample (small n) problems. Classical statistical methods and their extensions are not specifically designed for these challenging problems. Further, methods of analysis that are not designed with a fundamental understanding of the processes of tumorigenesis are likely to be misapplied. Everyone involved in cancer biomarker discovery and evaluation, including those involved in the bioinformatics of disease prediction modeling, should read the seminal Hanahan and Weinberg (2000) paper, and try to come to terms with heterogeneity in type and in molecular processes as a fundamental characteristic of human tumors.

“Appropriately designed”, like ‘easy to use’ has a dual meaning. Software development standards adopted and promoted by the Cancer Biomedical Informatics Grid (caBIG™) include formal ranking categories of the caBIG compliance criteria. As a cottage industry, bioinformatics has progressed without tight control over development standards. This often makes resource re-use difficult. As cancer centers collaborate to build a 100% open-source, grid-based system for cancer informatics, the adoption of development standards will help harmonize software and enhance interoperability. These important development standards need not impede progress in the determination of ‘appropriate design’ of the functionality of the software in any way.

Development efforts published in *Cancer Informatics* will not be restricted to those that are compliant according to the caBIG published criteria for compatibility, but if an application note describes a resource that has been designated as achieving the caBIG Silver-level compatibility, the authors are encouraged to inform the reader in the abstract.

**Robust:** robustness is a technical term that means that a method or model can be expected to perform well in a variety of contexts, with varying assumptions. Current published cancer microarray datasets do not meet the assumption of classical statistical methods (t-tests, for example); assumptions of normality (within-gene distributions) are not met, the sample sizes are too small, and while we have known for some time that cancer is a heterogeneous disease, we are just now beginning to understand how heterogeneous a given type or even subtype of cancer may be. In my own work I draw a distinction between ‘ecological models’ and ‘oncological models’. In the former, all variables apply to all individuals. In the latter, some variables are irrelevant for some patients in what may be a largely *unpatterned* manner. Advances in analysis are needed that anticipate the underlying heterogeneity that is characteristic of biomarker distributions among patients. This conceptual break has fostered the development of new methods for finding differentially expressed genes, and should yield novel insights and approaches for making classification inferences.

Robustness also has dual meaning in software performance. No doubt all of us have experience with software that is robust, or fault-tolerant, to varying degrees!

There are obviously large open areas of research in *Cancer Informatics*. We would hope to help set the stage for the final era of cancer research by holding our contributor’s work to the highest standards. Broadly speaking, accepted practices in biomedical informatics have yet to be distilled. For biomarker studies, however, a current of awareness of the need for standards is coursing through the cancer biomarker research community. Much of this discussion has been centered on concerns that data from proteomic biomarker discovery studies are not ‘reproducible’. Unfortunately, a major misunderstanding has proliferated throughout the cancer research community to the point where it is often stated that data from proteomics profiling studies are not reproducible.

This is patently false. Individuals who make such claims are actually confusing important key concepts. Consider the two statements:

- Data from proteomics technology *x* are not reproducible.
- Results from a proteomics study *y*, that happened to use technology *x*, have low generalizability due to poor study design.

Parsing these concepts it utterly essential to the promotion of good science around biomarker research in general. An effort should be made to expand the appreciation of the distinction between generalizability and reproducibility. Specifically, measurement reproducibility is a necessary but insufficient condition for generalizability.

Generalizability requires:

- accurate measurement (reproducibility)
- proper experimental design to protect against potential bias, sufficient sample sizes, and
- proper evaluation of algorithms/decision rules/prediction models.

It should also be remembered that measurement reproducibility (accuracy) of any technology is well assessed using self vs. self comparisons, but is not at all well assessed with studies designed to discover and assess the generalizability of learned classifiers. Authors of biomarker studies in general should be careful not to claim demonstrated reproducibility or generalizability unless they have been tacitly measured or estimated.

Such reproducibility studies have only begun for SELDI-TOF/MS or MALDI-TOF/MS, and are important whether the biomarker technology is destined for clinical adoption or not. Low measurement reproducibility can put a hard ceiling on the generalizable performance estimates that no amount of algorithm tuning can overcome – without overtraining. What number of technical replicates, *k* should be used as a standard practice remains an open question for most biomarker discovery technologies, and the cancer biomarker research community or technology vendors should undertake reproducibility studies that determine how laboratory workflows and protocols may be changed to increase measurement reproducibility should be undertaken and published by technology vendors.

Measurement reproducibility studies, if ever conducted, are often conducted well after an initial hefty investment in performance evaluation and biomarker discovery; they are usually conducted as intra-assay variability studies at the stage of translation. As new technologies are developed, it would appear to be incumbent upon early adopters, or even the companies who proffer new technologies to provide a thorough reporting of objectively conducted technical (measurement) reproducibility studies of their technology *before* the hefty investment is made by the academic and medical community. Secondary exploratory studies, and then evaluation studies, validation studies, and, finally, translation studies (clinical trials) of biomarker panels should not be limited by unoptimized, unrobust new protocols and technology. The adage ‘buyer beware of new high-dimensional biomarker technologies without sufficient up-front measurement reproducibility studies’ should be adopted by cancer centers. As new technologies are developed, false starts can be expensive – not just in terms of dollars, but also research person-years, career development, and, ultimately, patient’s lives.

Authors might consider identifying their biomarker studies as ‘Exploratory’; ‘Evaluation’; ‘Validation’; and, finally, ‘Translational’ so the reviewers and readers know how best to interpret the intent of the study and so authors need not feel compelled to over interpret their results. In the US Early Detection Research Network effort, studies are ranked according to pre-validation or discovery studies (Sullivan Pepe et al., 2001). Movement is underway at the EDRN/caBIG interface for the development of data elements designed to represent the most critical information from biomarker studies; the Standards for Reporting of Diagnostic Accuracy (STARD) initiative (Bossuyt et al., 2003) provides a valuable starting point. Researchers involved in biomarker studies will find within the STARD paper a useful checklist for their studies.

Reproducibility of prediction model construction (better termed ‘generalizability’) should be a first concern of exploratory studies; i.e., the ultimate validation (and demonstration of reproducibility) would be the discovery of the same decision rule at two sites given similar populations, or even the interchangeability of model parameters learned at various sites, but alas! cancer is unforgivingly complex, everything measured is measured with error, and potential bias factors abound in studies of diagnostic accuracy, but their general impact of those same studies in not at all well characterized (Whiting et al., 2004). In the absence of repeated studies, estimates the generalizability of the performance measures of a model should be considered as important as measurement reproducibility, and the two should not be confused.

As early as possible, ‘reproducibility’ studies should be performed to not only assess the reproducibility of measurements, but to assess the generalizability of disease prediction model. Ideally, studies should be repeated at another site on new patients as soon as possible hand the prediction models compared directly. Such independent confirmation studies should be given high funding priority and should not be viewed as mere ‘me-too’ proposals. The EDRN is developing standardized reference sets for use as blinded independent sample sets, upon which the generalizability of the models can be further tested.

Research on serum proteomic peptide profiling in cancer got off to an ‘odd start’, with an initial study reporting unrealistically high performance measures, arguably due to an error in control of experimental design (Baggerly, 2005, this volume). Reviewers should not hold the expectation that published results in all studies will achieve near-perfection. One highly relevant standard of performance reporting should be *improvement over existing clinical practice*. Funding decisions should not be made to further validate, or translate biomarkers, on the basis of failure to achieve SN = .99 and SP = 1.0, especially with preliminary data. Given the toll of cancer-related mortality, the importance of this should not be understated. Patients cannot wait for us to find the perfect biomarker, and it should be considered unethical to hold too high standard that prevents adoption of new markers that lead to real improvements in chances of survival by early detection of some of those 1500. Complicating consideration is whether a biomarker is being developed as a screen, in which case the SP should extremely high, or if the marker is being developed to be used in conjunction with current clinical practice. In screening programs, the requirement of high SP exists to avoid unnecessary instrusional follow-up procedures (biopsies, laproscopy) that come with risk. Moreover, screening markers that maximize NPV could lead to identification of individuals who are not likely to develop cancer, and therefore who may not require further screening. Clinical applications of biomarkers for specific groups of patients may occur more rapidly, and study designs and evaluation of generalizable performance estimates should be done in consideration of the specific intended purpose. Efforts to create screening tests in hope of massive consumption must be weighed against the very real need for and potential utility of markers or panels in the current clinical context. Research is needed on how to incorporate various types of clinicopathological/biomarker data sources to create generalizable composite indices or rules that allow for heterogeneity in the reporting performance of each individual source of information.

Considering the confusion that surrounds the problem of reproducibility vs. generalizability, I offer the following list of concerns for consideration as a minimum set of criteria for any study that aims to discovery, evaluate, validate, and/or translate a panel of cancer biomarkers:

1. Please read Dr. David Ransohoff’s contributions on the importance of avoiding bias (Ransohoff, 2005) and on rules of evidence (Ransohoff, 2004). Before running any samples, researchers might create a design matrix and examine it for potential confounding factors, randomize class assignment in treatment/controls studies, and randomize or interleave the sample processing order. Note that confounding factors only make any biomarker study indeterminant. This does not mean that all cancer signal is removed by the confounding factor; it only means that the authors cannot be certain which (if any) apparently diagnostic features are informative due to cancer and which may be due to the confounding factor. In my opinion, such studies should still be published if only to make the data available as independent test samples for more properly designed studies. Authors and reviewers should consider that confounded studies may or may not add value in terms of generalizable decision rules (only more test samples can tell), but the data can still be used as an independent test validation set. Such studies are welcome as technical report in *Cancer Informatics*, and authors should be certain to identify the factor that they consider to be confounding factors.
2. Please read Dr. Margaret Sullivan Pepe et al.’s important outline of phases of biomarker development (Sullivan Pepe et al., 2001) and subsequent contributions on the evaluation of cancer disease modeling, and understand the specific Phase(s) of biomarker development a particular project represents. Reviewers should avoid evaluating a biomarker discovery study as a biomarker validation study, and authors should avoid overdrawing conclusions beyond that which the study provides.
  If authors are ambiguous about the intended scope of their study, readers can be confused on whether the authors have overextended their conclusions. If a study is labeled “Exploratory”, then the reviewers and the readers can assess the completeness of the study – and therefore the conclusions drawn – in a more informed manner. This will not only allow researchers to share results that may be too preliminary for a complete study; it will also reduce ‘publication bias’; i.e., the potentially widespread practice of ‘resultsshopping’, and publication of only positive results. A study that uses one data set from only one site to learn and evaluate a classifier is likely (but not necessarily) to be an ‘Exploratory’ or discovery study. A study that uses data or a model from another site and data from a relatively small number of samples to evaluate the model is likely to be an “Evaluation” study. Studies that provide extensive use of new test samples or reference samples expressly for the purpose of confirming the expected performance of a set decision rule is a ‘Validation’ study. Surely the lines between these studies are blurry, and a given study may report more than one type of result. The intent is to allow specific knowledge claims by authors to be interpreted in the context of their self-identified scope of study, and prevent misunderstanding by readers who might consider conclusions to be overdrawn. Authors should strive to objectively represent their own research and avoid ‘publication bias’.
3. Absolutely, unequivocally, without fail, provide unbiased (or at least low-bias) estimates of the performance of the biomarker panel through the use of training and independent test sets (i.e., do not overtrain). There is never any reason not to perform objective, unbiased, externally generalizable evaluation. One sometimes must be diligent to avoid a design of analysis and models that leads to overtraining (especially with mid-dimensional panels of markers). It is essential that researchers understand that when any information on biomarkers (overexpressed vs. underexpressed), or parameters estimates are generated or even gleaned using a given data set, the generalizability is always limited, and, paradoxically, the more information gleaned from a single data set, the less generalizable the bulk of the information will tend to be for new cases. Happily, more generalizable information and parameter estimates can also nearly always be provided through carefully constructed study designs that employ training set/test sets. Sometimes it is easy to fall into a trap where model parameters only are gleaned from a data set that is intended for use as a validation test set, but the markers themselves are not learned from that validation test set. Such instances can be avoided by following the rule: if a decision rule required only gathering measurements from a test set, overtraining is not likely. If a decision rule requires model parameters estimated from a test set, then the research in still in the learning or evaluation mode, and not (exclusively) in the validation mode. The overt demarcation of exploration, evaluation, validation and translation studies (see above) can similarly help.
4. Describe the final decision rule(s) in sufficient detail to allow it to be applied directly to new test samples by other researchers. Unless a classifier or decision rule can be independently and readily applied by independent researchers, it is likely to go unvalidated. Generic performance reports of classifier performance may show potential of a technology or a data set, but others should be able to make predictions on new samples. This is best facilitated either by describing decision rules in the text of the paper and sharing source code.
  Open source initiatives such as the caBIG (http://cabig.nci.nih.gov/) have set the stage for setting standards for development practices, for sharing source code, and to facilitate data sharing. For the purpose of evaluating original research projects, *Cancer Informatics* will require authors to submit, as supplementary material, the source code for their applications, as well as an executable version, and sufficient documentation to allow anyone to reproduce their published results. Researchers will be expected to also make the data used available as supplementary material in an easily accessible format in which it is typically analyzed (.txt, .csv) for review, and for the data to be made available to the public within one year of the date of publication. Providing access to the source code used to discover and evaluate the panel of biomarkers and reporting which data were used as an independent test data set is essential for sufficient peer-review. *Cancer Informatics* will store source code and executable as supplementary material for open source projects, but authors should indicate a permanent repository for source code when one is available. Supplementary material might also include metadata that describes which options of analysis were used (perhaps in a machine and human-readable form, such as XML).
5. Provide a comprehensive description of the evaluation of alternative methods of analysis used in the learning stage, and the unbiased performance measures associated with the suboptimal methods.
  Critical to further evaluation of the markers with new prediction analytic methods is the ability to autotrack provenance of changes made to the data prior to analysis, to track analysis settings, and to use the same training/test set splits. Markup language can be generated as metadata that can record the random number series used to generate training/test set splits; a table of the training/test set/validation set labels published as supplemental material would allow anyone to directly and fairly compare new methods to those conducted in a given study. This is a high priority in the development efforts underway in the caBIG proteomics informatics group.
  If additional methods were applied beyond those used to produce the published results, their performance should ideally also be reported; if many approaches are tried to summarize in the text of the paper, their performance characteristics can be summarized as a supplemental table. Often the range of performance in the top 10% of models explored is of interest and can be reported in the results section of the study itself. An alternative approach is to produce a ‘data set ROC plot’, in which the generalizable performance measures (SN, 1-SP) of each model attempted are reported at location of the globally optimal performance (overall minimized classification error) of each method. It is potentially misleading to perform many disease prediction models and only report the overall best results.
6. Compare the performance of the panel to the reported generalizable performance of other proposed biomarkers or panels, and (importantly) to the performance characteristics of current practices for that particular cancer diagnosis, prognosis, or therapy efficacy/safety study problem for that particular cancer.
  Progress in molecular diagnostics should not be limited by the search for the perfect biomarker. Improvements over the performance of current clinical practice is an important benchmark; relative standing among an array of proposed biomarkers and predictions models should not be seen as competition at the time of publication; rather, effort that goes into summarizing the reported performance of markers and models can help in setting future directions of the use of combinations of proposed markers.
7. Whenever possible, apply the final optimized decision rule(s) to existing published data sets to provide additional evaluation and report performance regardless of their values.
  Published data sets provide opportunities to move a study from the ‘exploration’ or discovery stage into the ‘evaluation’ stage; use of a sufficient number of published data sets could also move a study from ‘evaluation’ into ‘validation’ stage. Concern over the use of common protocols in such studies may be overstated; after all, we would hope for panels that are robust to small changes in protocols and that are robust to site-to-site variability. Therefore, achieving consistently good performance across sites *in spite* of the use of different protocols should be considered more remarkable than achieving the same with one set of highly controlled laboratory protocols. JPL’s eCAS, VSIMS and ERNE resources (Crichton et al references; Tenenbaum, 2003) will act as a data repository for EDRN (http://www.compass.fhcrc.org/enterEDRN/) biomarker published studies, and efforts are underway to harmonize ongoing proteomics development efforts in the caBIG (http://cabig.nci.nih.gov/workspaces/Proteomics/) with these highly advanced resources.
8. Provide an error report for each patient (sample) to better inform the reviewer on which patient (samples) may be difficult to predict accurately, and examine the error report in light of the design matrix to uncover any unforeseen sources of variability. It is important to avoid then re-analyzing the data with a new perspective provided by a *priori* pattern detection; if such re-analyses are conducted, the results should be reported as hypotheses generated by pattern discovery, and not reported as hypotheses tested and found to be corroborated by a critical test;
9. Provide an estimate of the stability of generalizable performance characteristics over a range of sampling effort (N); this ‘variance of performance analysis’ is an empirical substitute for power analyses and informs the reader on both the sufficiency of the experimental design (sufficient sampling effort) and on the inherent variability associated with the sampling effort.
  Power analysis is extremely tenuous in biomarker discovery, evaluation, and validation with high-dimension analysis. In addition to N, the sample size, power is a function of (a) the effect size, which is not known or estimable in the discovery phase, (b) the degree of random variance among variables (balanced study designs are more efficient than randomized studies), (c) the stringency of a test or criterion used for identifying potential biomarkers (under some methods, stringency itself is optimized to minimize the false discovery rate), and, of course, (d) the type of test used. Even if preliminary data are available, one can’t perform power analysis per se because the true biomarkers for a given study problem are not known.
  Given preliminary data, one can, however, measure and report the response in variance of unbiased, externally generalizable (test set) performance characteristics to increasing sample sizes (e.g., test set SN, SP, and ACE (= 1-accuracy)). N can be considered sufficient when the variance ratio between a doubling of N is near 1.0. These “variance of performance” analyses could be considered in lieu of power analysis. The variance in the test score for the genes or peptides found to be significant using all of the available samples can be similarly plotted, but as the result is based on the overall data set, a biased result is possible.
10. Because we presumably don’t yet know all of the dimensions of clinically relevant cancer subtypes, the distinction between supervised and unsupervised classification is blurry in cancer genomics and proteomics studies. Supplemental data should perhaps include not only the range of performance of the top 10% of the models explored, but also a report of the class prediction errors for each sample. This is good practice for proper consideration of the reported classifier performance measures. If a subset of samples is consistently misclassified, this may inform the researcher about their degree of understanding of unmeasured sources of variability, and may provide direction for re-analysis, or for further study. Identifying patient subgroups first with unsupervised clustering, followed by a post-hoc search for factors that may explain those subgroups is risky due to the possibility that if one searches for such factors long enough, one or more ‘interesting’ subgroups will be found; nevertheless, such an exercise could prove invaluable to the detection of unseen, incidentally partial or complete confounding and should be done prior to interpretive analysis. Training/test set analysis designs are essential to protect against overinterpreting such emergent patterns.
11. Re-consider surrogate biomarkers examined with good science. Other journals may be more suitable for technology improvements (e.g., *Clinical Chemistry; Biotechniques*); still others more appropriate for the biomarker discovery phase (e.g., *Disease Markers; Cancer Genomics and Proteomics*); and clinically oriented journals may be best suited for biomarker validation studies (e.g., *Journal of Clinical Epidemiology; Clinical Cancer Research*). Bioinformatics and biostatistics combined are so critical to the biomarker evaluation phase that this journal would hope to attract the bulk of cancer biomarker/prediction model evaluation studies (in addition to the broad scope of focus represented in our Editorial Board!). Studies (especially multisite validation studies) that provide unbiased estimates of performance that improve over other proposed and existing markers and models, but that may not have identified all of the markers in the panel or understand their function (so-called ‘surrogate markers’), may be considered by most to be ‘evaluation’ or even ‘discovery’ studies; either way, they are very welcome in *Cancer Informatics*. To the cancer biomarker research community in general, I pose the following question: can patients whose survival may be improved by early detection afford our need to know the identities and functions of all of the markers on a panel, or should we also proceed as rapidly as good science allows, to answer questions regarding the performance characteristics of classifiers based on markers, identified or unidentified, content to know that we will eventually know the identify of markers that are highly predictive or prognostic? This issue should be debated at the highest levels with patient advocates and program officers taking a close look at the criteria being applied to fund cancer biomarker discovery, evaluation, and validation studies. If a study has been performed in a manner that demonstrates that a panel of biomarkers performs better than current practice on two independent test validation sets (data from new patients collected by researchers at two independent sites), and a follow-up study demonstrates that the biomarkers are sufficiently specific for a cancer, or even ‘for cancer’, then perhaps the biomarkers and the prediction models have made their case, and the inevitable identification and functional characterization can wait. Studies on computational resources that improve the ability of researchers to perform peptide or protein ID from spectra are of course also very welcome in *Cancer Informatics*.

Any or all of the points raised in the above proposed critical set of standards for excellence for cancer biomarker research are open to debate, as they represent an amalgam of perspectives that will no doubt evolve as we continue to learn from each other. *Cancer Informatics* will be, I hope, a forum that promotes active and productive consideration of these and other topics to the end of helping to resolve the cancer problem as soon as possible.
